# *N-*acyl-homoserine lactones-producing bacteria protect plants against plant and human pathogens

**DOI:** 10.1111/1751-7915.12177

**Published:** 2014-09-19

**Authors:** Casandra Hernández-Reyes, Sebastian T Schenk, Christina Neumann, Karl-Heinz Kogel, Adam Schikora

**Affiliations:** Institute of Phytopathology and Applied Zoology, IFZ, Justus Liebig University GiessenHeinrich-Buff-Ring 26-32, Giessen, 35392, Germany

## Abstract

The implementation of beneficial microorganisms for plant protection has a long history. Many rhizobia bacteria are able to influence the immune system of host plants by inducing resistance towards pathogenic microorganisms. In this report, we present a translational approach in which we demonstrate the resistance-inducing effect of *Ensifer meliloti* (*Sinorhizobium meliloti*) on crop plants that have a significant impact on the worldwide economy and on human nutrition. *Ensifer meliloti* is usually associated with root nodulation in legumes and nitrogen fixation. Here, we suggest that the ability of *S. meliloti* to induce resistance depends on the production of the quorum-sensing molecule, oxo-C14-HSL. The capacity to enhanced resistance provides a possibility to the use these beneficial bacteria in agriculture. Using the *Arabidopsis*-*Salmonella* model, we also demonstrate that the application of *N*-acyl-homoserine lactones-producing bacteria could be a successful strategy to prevent plant-originated infections with human pathogens.

## Introduction

The best understood mechanism of systemic resistance induced by beneficial microorganisms is the induced systemic resistance (ISR), where plants have a potentiated defensive capacity against future biotic challenges. Its mechanism requires the presence of an operable non-expressor of PR1 (NPR1) and components from ethylene (ET) and jasmonic acid (JA) signalling cascades. Together with systemic acquired resistance (SAR), which is usually associated with a previous pathogen attack, ISR and SAR are under intense study (Van Wees *et al*., 2008; Dempsey and Klessig, 2012; Fu and Dong, 2013; Shah and Zeier, 2013). Nevertheless, the molecular basis of ISR is not completely understood because, for example, the beneficial *Pseudomonas fluorescens* strain 89B61 induces resistance in a JA- and ET-independent manner (Ryu *et al*., 2003).

The exchange of signals between plants and nearby rhizobacteria contributes to the activation of ISR. Small signalling molecules, for example *N-*acyl-homoserine lactones (AHLs) from many Gram-negative bacteria, are used for their intra-population communication called quorum sensing (QS) (Kaplan and Greenberg, 1985; Fuqua and Winans, 1994). Remarkably, plants are able to detect and respond to bacterial QS molecules (Mathesius *et al*., 2003). The detection of AHLs and systemic response is an essential aspect of the establishment of mutualistic relationships (Bauer *et al*., 2005). Studies of plant responses to AHLs were first done in the model plant *Medicago truncatula*, where these molecules were found to affect extensive functions including cytoskeletal elements, transcriptional regulation and responses to defence, stress and hormones (Bauer *et al*., 2005). Another study on the interaction between *Serratia liquefaciens* and tomato (*Solanum lycopersicum*) provided also indications that QS molecules of rhizosphere bacteria influence plant defence responses (Schuhegger *et al*., 2006). In this study, authors used the *S. liquefaciens* strain MG1, which produces C4- and C6-homoserine lactones when colonizing the root surface (Gantner *et al*., 2006). Colonization of the roots with *S. liquefaciens* MG1 protected tomato plants against the leaf-pathogenic fungus *Alternaria alternate*, in contrast to the AHL-negative *S. liquefaciens* mutant MG44 that was not able to provide such protection (Schuhegger *et al*., 2006). Similarly, colonization with the AHL-producing *Serratia plymuthica* strain HRO-C48 protected cucumber plants (*Cucumis sativus*) from the damping-off disease caused by *Pythium aphanidermatum*, as well as tomatoes and beans (*Phaseolus vulgaris*) from the infection with the grey mould-causing fungus *Botrytis cinerea* (Pang *et al*., 2009). Comparable with the previous study, the AHL-negative *splI*^-^ mutant of *S. plymuthica* could not confer protection against both pathogens. Moreover, the resistance induced by *Ensifer meliloti* (*Sinorhizobium meliloti*) against *P. syringae* in *Arabidopsis* plants was depended on AHL accumulation (Zarkani *et al*., 2013). These results provided indications that AHLs play a role in the modulation of the plant immune system. Opposite results were reported for *Arabidopsis thaliana*, where *S. liquefaciens* MG1 and its AHL-negative mutant MG44 induced similar resistance against the pathogenic bacterium *Pseudomonas syringae*, suggesting an AHL-independent effect (von Rad *et al*., 2008).

In addition, the application of commercial AHLs also had an impact on plant physiology. AHL application induced changes in gene expression, altered protein profiles, modified root development and enhanced resistance against bacterial and fungal pathogens. This effect leans on a stronger and prolonged activation of MPK6 (Mathesius *et al*., 2003; Ortiz-Castro *et al*., 2008; von Rad *et al*., 2008; Schikora *et al*., 2011a,b; Bai *et al*., 2012; Schenk *et al*., 2012). Furthermore, plant responses to different AHL molecules appear to be AHL specific. Proteome analysis revealed around 150 differentially accumulated proteins in response to the application of either the commercial oxo-C12-HSL or the oxo-C16:1-HSL isolated from a *Sinorhizobium meliloti* culture (Mathesius *et al*., 2003). Correspondingly, an application of three commercial AHLs (C6-HSL, oxo-C10-HSL, and oxo-C14-HSL) revealed specific transcriptional responses, depending on the length of the AHL molecule (Schenk *et al*., 2014). Interestingly, after exposure to the resistance-inducing oxo-C14-HSL and a further pathogen challenge, the plants expressed an increased accumulation of phenolic compounds, lignin and callose depositions in plant cell walls (Schikora *et al*., 2011a,b; Schenk *et al*., 2012; Zarkani *et al*., 2013). Additionally, accumulation of oxylipins in distal tissues promoted stomatal closure, thus enhancing plant resistance to bacterial infection (Schenk *et al*., 2014).

In this report, we present a translational approach in which the resistance-inducing effect of oxo-C14-HSL-producing *S. meliloti* strain *expR+* on *Arabidopsis* was verified in crop plants. We show that this effect depends on the presence of AHL molecules, because the inoculation of plants with the AHL-negative *S. meliloti* strain *attM*, which expresses a lactonase that inhibits the accumulation of AHLs, had no consequences on the plant resistance towards the tested pathogens. We used three different crop plants, which have significant impact on the worldwide economy as well as on human health and nutrition. In addition to the plant protective action, using the *Arabidopsis*–*Salmonella* model, we demonstrate that the use of AHL-producing bacteria could be a successful method to prevent plant-originated infections with human pathogens.

## Results

### Barley and wheat can be primed by *S. meliloti* *expR+* for enhanced resistance

**Fig 1:**
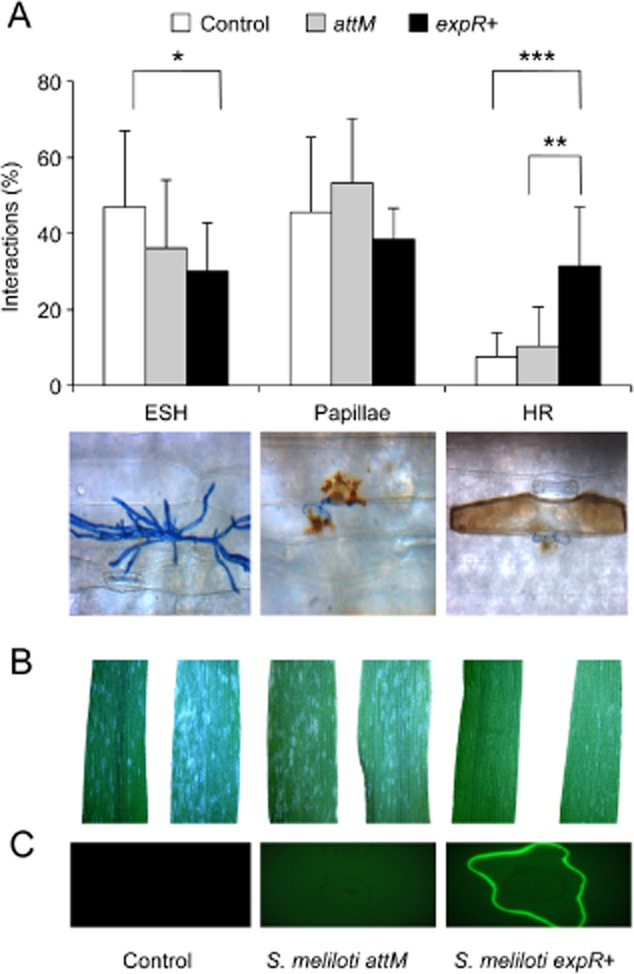
Oxo-C14-HSL produced by *S. meliloti* induced resistance against *B. graminis* in barley.A. Barley cv. Golden Promise plants were pretreated three times during 2 weeks with MgSO_4_ (control), the lactonase-expressing strain *S. meliloti* *attM* or the *S. meliloti* *expR+* strain, which produces significant amount of oxo-C14-HSL, prior to inoculation with the powdery mildew causing fungus *Blumeria graminis* f. sp. *hordei*. The percentage of interaction sites resulting in elongated secondary hyphae (ESH) demonstrating susceptibility against the pathogen, papillae or hypersensitive response (HR), both indicating resistance, was assessed 2 days after inoculation. *P ≤ 0.05; **P ≤ 0.005; ***P ≤ 0.0005 in Student's *t-*test. Experiment was repeated three times. Below the x-axis exemplary photographs are shown, presenting the possible results of interaction between barley leaf cells and *B. graminis* counted in A. From left: the formation of ESH, papillae and HR.B. *Blumeria graminis* mycelia developing on barley leaves 5 dai. Plants were treated like in A, representative photographs were taken using a standard binocular.C. Detection of oxo-C14-HSL produced by the *S. meliloti* strains used in A, using the biosensor bacterium *Escherichia coli* strain MT102.

Based on the observation that the inoculation of plants with AHL-producing bacteria induce resistance in plants towards diverse pathogens (Schuhegger *et al*., 2006; Pang *et al*., 2009; Zarkani *et al*., 2013), we tested the hypothesis that the induced resistance caused by *S. meliloti* in crop plants depends on AHL production in a similar way as in *A. thaliana* (Zarkani *et al*., 2013). For this purpose, we used two *S. meliloti* strains, the *expR+* strain carrying the pWBexpR plasmid (M. McIntosh, pers. comm.), which allows the production of the long-chain oxo-C14-HSL, and the *S. meliloti attM* strain that is unable to accumulate AHLs due to the expression of the *Agrobacterium tumefaciens* lactonase gene *attM* from the pBBR2-attM plasmid, (Zarkani *et al*., 2013). Barley cultivar Golden Promise plants were grown on soil and inoculated with *S. meliloti* by watering three times during 2 weeks before the challenge with the powdery mildew fungus *Blumeria graminis* f. sp. *hordei*. The cultivar Golden Promise is susceptible to *B. graminis*, i.e. 50% of its epidermal cells allow fungal penetration causing the formation of elongated secondary hyphae (ESH) and subsequent disease symptoms (Fig. 1A and B). However, plants inoculated with the oxo-C14-HSL-producing *S. meliloti* strain *expR*+ (Fig. 1C) showed enhanced resistance as a result of the augmentation of hypersensitivity response (HR) reactions at the sites of fungal penetration, thus diminishing the number of developing pustules (Fig. 1B). Correspondingly, the lack of this enhanced HR response in plants inoculated with the *attM* strain (Fig. 1A) suggests that the increased resistance depends on the production of oxo-C14-HSL by *S. meliloti*.

**Fig 2:**
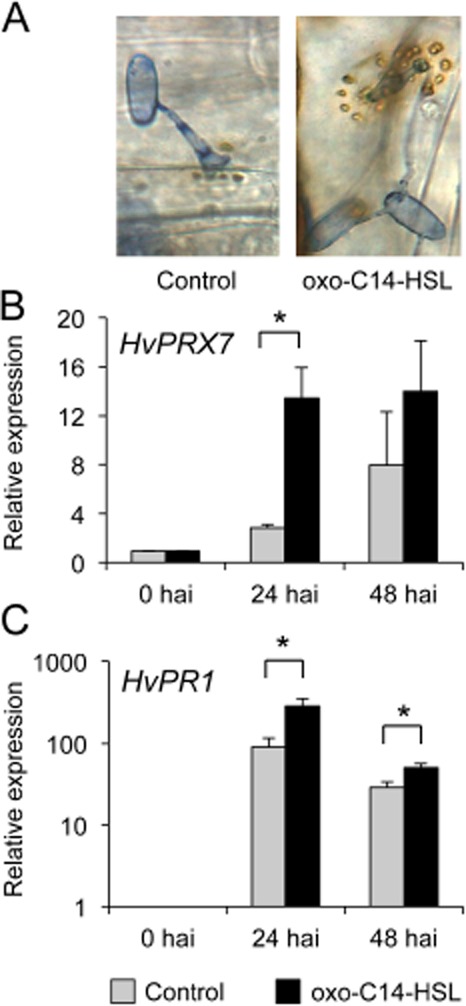
In oxo-C14-HSL-pretreated barley plants, papillae formation is associated with expression of *Peroxidase 7* and *Pathogenesis Related 1*. Sterile-grown barley cv. Golden Promise plants were pretreated with oxo-C14-HSL for 3 days prior to inoculation with *Blumeria graminis* f. sp. *hordei*.A. Formation of papillae in oxo-C14-HSL-pretreated and control plants on sites of attempted penetration by *B. graminis*. Image was taken 48 h after inoculation (hai) with *B. graminis*.B–C. Relative expression of *HvPRX7* (B) and *HvPR1* (C) in control and oxo-C14-HSL-pretreated plants assessed in hai as indicated. Expression values were normalized to the expression of *HvUBQ60* and 0 hai time point. *P ≤ 0.05 in Student's *t*-test. Experiment was repeated three times.

**Fig 3:**
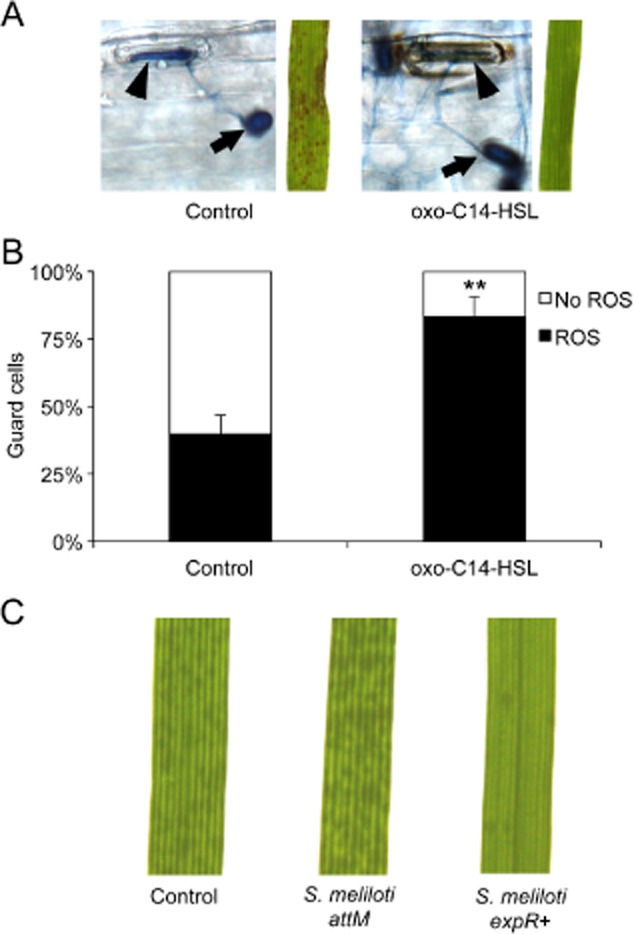
Enhanced accumulation of hydrogen peroxide in wheat guard cells after pretreatment with oxo-C14-HSL.A. Control leaves showing urediniospore (arrow) germination with directional growth of the hyphae towards stomatal opening (arrowhead) (control). Pathogen interaction with guard cells of oxo-C14-HSL pretreated leaves results in a significant accumulation of H_2_O_2_ (oxo-C14-HSL). Sterile-grown wheat cv. Bobwhite plants were pretreated with AHL solvent (control) or oxo-C14-HSL. Subsequently, leaves were inoculated with *Puccinia graminis* f. sp. *tritici*. DAB staining was performed 2 days after inoculation. On the right: exemplary leaves at 11 dai showing the differences in pustules development between control and oxo-C14-HSL-treated plants.B. The percentage of guard cells with higher accumulation of H_2_O_2_ as shown in A, 2 dai with *P. graminis*. **P ≤ 0.005 in Student's *t-*test. Experiment was repeated four times.C. Pustules development on wheat plants grown on soil for 10 days and inoculated three times with MgSO_4_ (control), *S. meliloti* *attM* or *S. meliloti* *expR+*. Representative images were taken 5 dai with *P. graminis* using a standard binocular.

Previously, we observed enhanced formation of papillae in barley plants pretreated with oxo-C14-HSL, which play a crucial role in resistance against fungal pathogens like *B. graminis* (Schikora *et al*., 2011a). Papillae are a complex structure between the plasma membrane and the plant cell wall, and depending of the plant species, the composition of these defence structures can consist of phenolics, reactive oxygen species (ROS) and cell wall proteins and polymers (Voigt, 2014). Hydrogen peroxide, one form of ROS that accumulates in forming papillae, is used by peroxidases to cause the cross-linking of proteins and phenolics for cell wall reinforcement. (Fig. 2A) (Hückelhoven, 2007; Deepak *et al*., 2005). In order to test whether oxo-C14-HSL influences this defence mechanism, we assessed the expression of one of the key enzymes in ROS production in barley, the peroxidase *HvPRX7*. To this end, barley plants were grown under sterile cultures for 10 days and the roots were pretreated with 6 μM oxo-C14-HSL or with the solvent control (acetone) for 3 days; subsequently, the first and second leaves were inoculated with *B. graminis*, and finally harvested for total RNA extraction after 24 and 48 h. Results from the quantitative reverse transcription polymerase chain reaction (RT-PCR) revealed that in contrast to control, plants pretreated with oxo-C14-HSL displayed a higher expression of *HvPRX7* in response to *B. graminis* at 24 hai (Fig. 2B). Similarly, the expression of the *Pathogenicity Related1* (*HvPR1*) gene was higher in oxo-C14-HSL pretreated plants, compared with the control (Fig. 2C). To substantiate our findings, we tested the impact of oxo-C14-HSL on ROS production by exploiting a different pathogen–host system. We used wheat plants cultivar Bobwhite grown and pretreated as described above before a challenge with the stem rust*-*causing fungus *Puccinia graminis* f. sp. *tritici*. Because *P. graminis* enters the interior of mesophyll tissues via stomata openings, the closure of stomatal pores is an effective protection mechanism against this fungus (Fig. 3A). In addition, guard cells of plants pretreated with oxo-C14-HSL presented an enhanced accumulation of H_2_O_2_, as indicated by the positive 3,3′-diaminobenzidine (DAB) staining (Fig. 3A and B). The disease symptoms observed on leaves at 11 days after inoculation were consistent with ROS accumulation (Fig. 3A, images on the right). These results suggested that oxo-C14-HSL primed barley and wheat plants for enhanced ROS production after a challenge with the pathogens. In the same manner, the oxo-C14-HSL-producing *S. meliloti* strain *expR+* conferred protection against *P. graminis* in wheat plants (Fig. 3C). In comparison with the control and treatment with the *S. meliloti attM* strain wherein a high development of fungal pustules 5 dai was observed, *S. meliloti expR+*-treated plants presented lower number of developing pustules on leaves, suggesting that in analogy to barley (Fig. 1) and *Arabidopsis* (Zarkani *et al*., 2013), oxo-C14-HSL-producing bacteria protected wheat plants against *P. graminis*.

### Inoculation with oxo-C14-HSL-producing *S. meliloti* protects tomato from late blight disease

**Fig 4:**
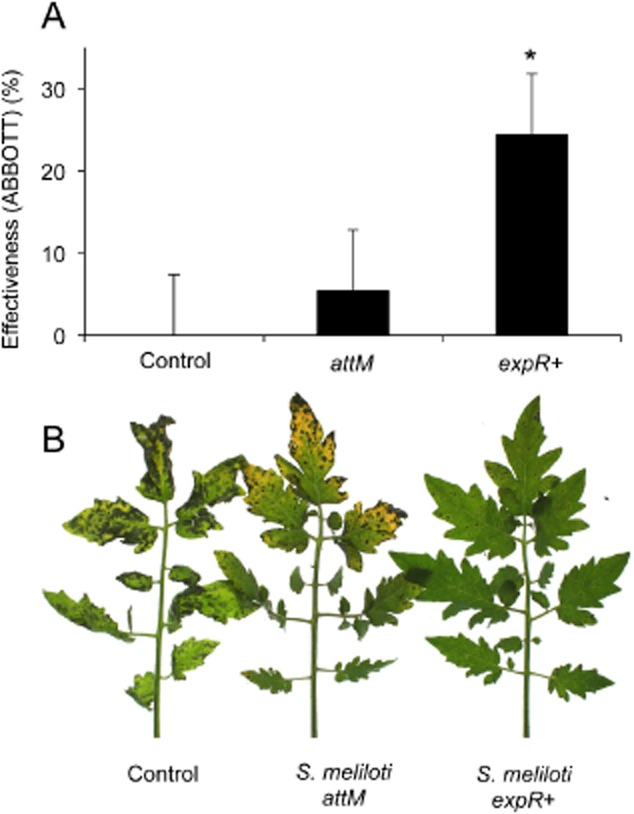
Treatment with *S. meliloti* strain producing oxo-C14-HSL increases resistance against late blight in tomato. Roots of tomato cv. Moneymaker plants were inoculated via watering with the oxo-C14-HSL-producing *S. meliloti* strain *expR+*, the *attM* lactonase-expressing *S. meliloti* *attM* strain or MgSO_4_ (control) for 4 weeks prior to inoculation with the oomycete *Phytophthora infestans*. Disease symptoms were assessed 1 week after inoculation. Data represent mean from three independent repetitions.A. Efficiency of treatment assessed using the Abbott formula. *P ≤ 0.05 in ANOVA.B. Macroscopic symptoms caused by the late blight agent *P. infestans* on tomato plants.

We extend our analysis from monocots to a dicot crop plant with high agronomic interest. We tested the induced resistance in tomato against *Phytophthora infestans*, which is nowadays one of the principal pathogens causing the late blight disease and a worldwide damage of 6 billion US dollars each year (Nowicki *et al*., 2011). Similar to the experiments with barley, soil-grown tomato plants cultivar Moneymaker were inoculated four times with *S. melioti* during 4 weeks prior to the challenge with *P. infestans*; control plants were pretreated with MgSO_4_. Disease symptoms were assessed 4 and 7 days after the challenge with *P. infestans* and the efficacy of the pretreatment was calculated using Abbott's formula (Abbott, 1925). In accordance to previous results, inoculation with *S. meliloti* strain *expR*+ induced resistance against *P. infestans* in tomato plants (Fig. 4A and B). Moreover, we observed differences between the inoculation with the oxo-C14-HSL-producing *expR*+ strain and the AHL-negative *attM* strain (Fig. 4C), implying that as in the case of fungal pathogens, resistance towards this *Oomycete* depends on the production of AHL.

### AHL-producing bacteria can promote resistance towards human pathogens

**Fig 5:**
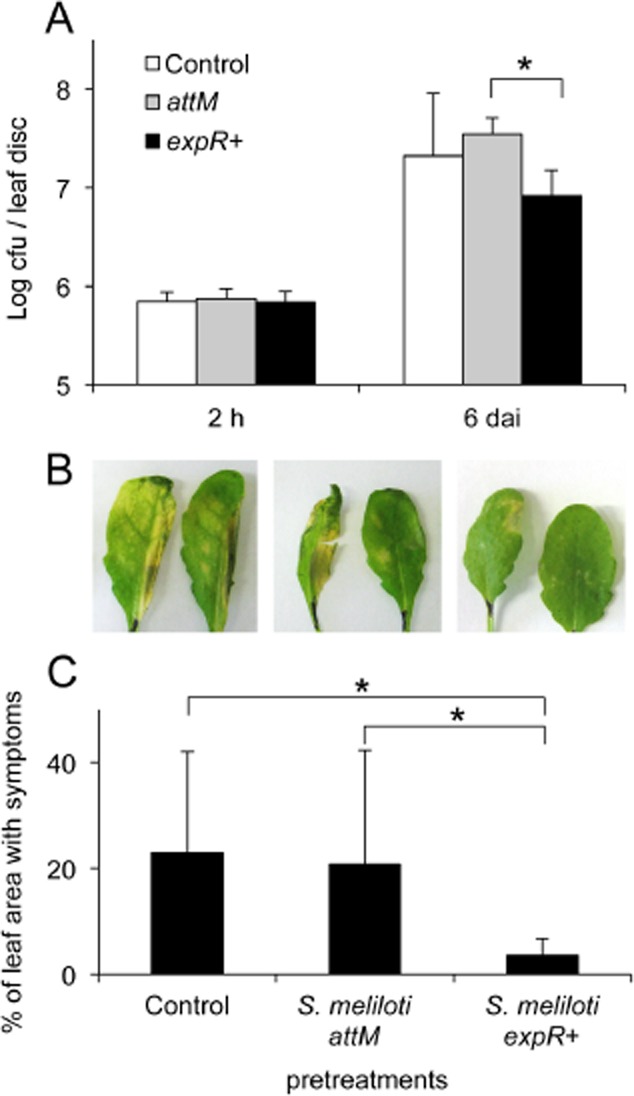
Treatment with oxo-C14-HSL-producing *S. meliloti* strain enhances resistance against the human pathogen *Salmonella enterica* serovar Typhimurium in *Arabidopsis*. Roots of *Arabidopsis* Col-0 plants were inoculated via watering with the oxo-C14-HSL-producing *S. meliloti* strain *expR+*, the *attM* lactonase-expressing *S. meliloti* *attM* strain or MgCl_2_ (control) four times during 4 weeks prior to syringe infiltration of leaves with *Salmonella* Typhimurium bacteria.A. Proliferation of *Salmonella* Typhimurium in *Arabidopsis* leaves assessed at 2 h and 6 days after inoculation (hai and dai respectively). *P ≤ 0.05 in Student's *t*-test. Data present a mean from three biological repetitions.B. Macroscopic symptoms caused by *Salmonella* Typhiumurium on *Arabidopsis* leaves.C. Quantification of symptoms caused by *S*. Typhimurium on *Arabidopsis* laves was performed using the algorithm described in (Schikora *et al*., 2012). *P ≤ 0.05 in Student's *t*-test.

*Salmonella* are Gram-negative bacteria that are able to colonize humans and plants. These bacteria are the causal agents of gastroenteritis and typhoid fever in humans due to the ingestion of contaminated food or water (Pang *et al*., 1995). Additionally, in recent years the proportion of raw-food related outbreaks in the USA reached 25% (Rangel *et al*., 2005). The increasing number of infections related to the consumption of fresh fruits and vegetables contaminated with these bacteria is very alarming and suggests that plants may be a substantial reservoir for *Salmonella*. Many reports proposed a complex interaction between *Salmonella* and the host plant because the plant immune system seems to play a key role in the outcome of the colonization (Schikora *et al*., 2011b; 2012; Shirron and Yaron, 2011). For this reason, the induction of defence mechanisms by oxo-C14-HSL-producing *S. meliloti* was tested as a potential measure to reduce the risk of plant-related infections. According to the experiments above, soil-grown *A. thaliana* Col-0 plants were watered four times with *S. meliloti* or with the respective controls before defying the plants with *Salmonella enterica* serovar Typhimurium strain 14028s. The proliferation of *S*. Typhimurium was assessed during 6 days after syringe infiltration. Interestingly, *Arabidopsis* plants pretreated with the *S. meliloti expR+* caused a lower *Salmonella* proliferation than plants pretreated with *S. meliloti attM* or MgCl_2_ (Fig. 5A), which corresponds to the diminished disease symptoms in leaves from *S. meliloti expR+* pretreated plants (Fig. 5B and C). This suggest that in line with the effects seen in barley and tomato, the production of AHLs allowed *S. meliloti* to prime *Arabidopsis* plants for an enhanced defence against *Salmonella*.

## Discussion

In this report, we present the impact of oxo-C14-HSL-producing bacteria on the plant immune system. We demonstrated that the previously described AHL-priming (Schenk *et al*., 2014) and the effect of oxo-C14-HSL-producing bacteria is not restricted to the commercial molecule, nor to the model plant *A. thaliana* (Zarkani *et al*., 2013). In a translational approach, we showed that the use of AHL-producing bacteria could be a potential method to improve plant resistance and to decrease the yield loss caused by many pathogens. Moreover, AHL-induced resistance may reduce the risk of plant-originated outbreaks of salmonellosis in addition to other possible related diseases.

AHLs used by Gram-negative bacteria may vary in the length of the lipid side chain and in the substitution of the C_3_-atom (O- or OH- group). The length of the lipid side chain is essential for the effect on plants; for example, C4-HSL, C6-HSL, oxo-C6-HSL and oxo-C8-HSL promoted growth in *Arabidopsis* (von Rad *et al*., 2008; Liu *et al*., 2012; Schenk *et al*., 2012), whereas oxo-C10-HSL induced the formation of adventitious roots in mung beans (Bai *et al*., 2012). On the other hand, only some AHL molecules were reported to have resistance-inducing attributes. A comparison of plant responses with different AHLs at the transcriptome and proteome levels revealed that just long-chain AHLs could induce resistance-related changes at the transcriptome and proteome levels (Mathesius *et al*., 2003; Miao *et al*., 2012; Schenk *et al*., 2014). The molecule oxo-C14-HSL and to a lesser extend OH-C14-HSL induced resistance in *Arabidopsis* and barley plants towards biotrophic and hemibiotrophic pathogens (Schikora *et al*., 2011a,b). *Sinorhizobium meliloti* produces different long-chain AHLs, like oxo-C14-HSL (Teplitski *et al*., 2003; Zarkani *et al*., 2013), and therefore we decided to use this bacterium in this work to study the interaction between crop plants and AHL-producing rhizobacteria. Besides the acknowledged benefit that the interaction between *S. meliloti* and its native host *M. truncatula* results in nodulation and N_2_-fixation, the oxo-C14-HSL-producing *S. meliloti* strain induced resistance in the non-host plant *Arabidopsis* against *Pseudomonas syringae* pathovar *tomato* (Zarkani *et al*., 2013). For this reason, to ascertain our translational approach, we used economically important non-host crop plants of *S. meliloti* to study the impact of AHLs. The resistance induced by beneficial bacteria is referred as ISR, and it has been exhaustively studied employing *P. fluorescens* and *Bacillus* spp. bacteria; for review, see Pieterse and colleagues (2014). Today, the mechanism of ISR is relatively well understood; it requires NPR1 and components of the JA- and ET-signalling pathways. The transcription factor MYB72 was postulated to play a key role in ISR and link JA- and ET-signaling pathways (Van der Ent *et al*., 2008). However, the AHL-induced resistance, termed AHL priming, seems to depend on other mechanism. Resent findings indicated that instead of MYB72 and JA/ET pathway(s), the salicylic acid/oxylipin pathway influenced the AHL priming (Schenk *et al*., 2014). Moreover, the resistance-inducing effect of the long-chain AHLs in *Arabidopsis* was reflected in the reinforcement of the cell wall through the accumulation of callose, phenolic compounds and lignins, as well as to an intensified stomatal closure in response to bacterial attack. Likewise, we observed that the inoculation with oxo-C14-HSL-producing *S. meliloti* strain, as well as pretreatment with the pure oxo-C14-HSL molecule, primed barley and wheat plants for enhanced ROS production. Membrane-bound NADPH oxidase and apoplastic peroxidase proteins usually contribute to this transiently increased production of toxic ROS known as oxidative burst. In addition, ROS act as secondary messengers, which allocates them a central role in plant defence mechanisms (Marino *et al*., 2012).

Intriguingly, *S. meliloti expR+* induced plant resistance towards *Salmonella*, which is generally considered an animal or human pathogens. Until now, the infection mechanism(s) used by *Salmonella* to successfully and simultaneously colonize diverse hosts like animals and plants are poorly understood. Stomata openings were identified as possible entry points of bacteria into the inner layers of the mesophyll (Kroupitski *et al*., 2009). Remarkably, although some plant species (e.g. arugula) allowed *Salmonella* to internalize, others (e.g. parsley) seemed to prevent internalization (Golberg *et al*., 2011). The plant immune system appears to play a central role during colonization of *Salmonella* as indicated by the induction of defence mechanisms after inoculation with these bacteria (Schikora *et al*., 2008; Meng *et al*., 2013; Garcia *et al*., 2014) and by the fact that *Salmonella* can actively suppress those mechanisms in tobacco (*Nicotiana tabacum*) and *Arabidopsis* plants (Schikora *et al*., 2011a,b; Shirron and Yaron, 2011). Accordingly, the use of beneficial bacteria with the ability to enhance defence mechanisms in crop plants that are susceptible to infection with human pathogens could be an alternative to lower the risk of disease outbreaks associated with contaminated fruits or vegetables.

## Experimental procedures

### Plant growth

Barley (*Hordeum vulgare*) cultivar Golden Promise, wheat (*Triticum aestivum*) cultivar Bobwhite and tomato (*S. lycopersicum*) cultivar Moneymaker were grown on soil (for pathogenesis assays) or under sterile conditions (for transcriptional analyses and oxo-C14-HSL treatments) in a long day photoperiod at 19°C (barley and wheat plants) or at 25°C, 80% humidity (tomato plants). For the sterile system, 1 l of jars were used and plants grew on partially solidified 1/10 strength plant nutrient medium (PNM) (0.5 mM KNO_3_, 2 mM MgSO_4_, 0.2 mM Ca(NO_3_)_2_, 0.43 mM NaCl, 0.14 mM K_2_HPO_4_, 2 ml l^−1^ Fe-EDTA [20 mM FeSO_4_, 20 mM Na_2_EDTA]). *Arabidopsis thaliana* Colombia-0 plants were grown on soil at 22°C with 150 μmol m^−2^ s^−1^ light in 8/16 h day/night photoperiod.

### Seed disinfection

For sterile growth, barley (*H. vulgare*) cv. Golden Promise and wheat (*T. aestivum*) cv. Bobwhite seeds were soaked shortly in sterile water and then in 70% ethanol. Subsequently, the seeds were immersed for 90 min in 6% sodium hypochlorite with continuous stirring. Seeds were then rinsed two times with sterile water at pH 3.0 and several times with sterile water at pH 7.0 until no trace of sodium hypochlorite was detected. For germination, the seeds were placed on wet sterile filter paper for 3 days. *Arabidopsis thaliana* Col-0 seeds were surface-disinfected with 50% ethanol/0.5% Triton X-100 for 30 min and briefly rinsed with 95% ethanol. For germination, seed were placed for 10 days on sterile half-strength MS medium supplied with 0.4% gelrite and 1% sucrose.

### Oxo-C14-HSL treatment

Sixty millimolar stock solution of oxo-C14-HSL (Sigma-Aldrich) was prepared by dissolving the molecule in acetone. Ten-day-old barley or wheat plants cultivated on 1/10 PNM medium under sterile conditions were treated with oxo-C14-HSL at final concentration of 6 μM. All experiments were performed with the solvent control acetone.

### *Sinorhizobium meliloti* inoculation

*Sinorhizobium meliloti* (*Ensifer meliloti*) Rm2011 *expR+* containing the pWBexpR plasmid (M. McIntosh, pers. comm.) and *S. meliloti* (pBBR2-attM) carrying the lactonase gene *attM* from *Agrobacterium tumefaciens* were used. The rhizosphere was inoculated with *S. meliloti expR+*, *S. meliloti attM* (both OD_600_ = 0.2) or watered with 10 mM MgSO_4_ as control. Extraction of AHLs originated from *S. meliloti* liquid cultures was performed by vortexing with CHCl_3_ and discarding the aqueous phase after centrifugation. The CHCl_3_ phase was then evaporated using an ultra-speed vacuum centrifuge. The remaining residue was dissolved in acetone. Detection of oxo-C14-HSL was accomplished by dropping 10 μl of the extracted AHLs onto reporter bacteria: *Escherichia coli* strain MT102 carrying the pJBA89 plasmid [Ap^r^; pUC18Not-*luxR*-*P_luxI_*-RBSII-*gfp*(ASV)-T_0_-T_1_] (Andersen *et al*., 2001). After 2 h, the fluorescence was observed using an ex: 480/40 nm and em: 510-nm filters.

### *Blumeria graminis* treatment

Three days after the last treatment with *S. meliloti* strains or MgSO_4_, barley leaves (cv. Golden Promise) were inoculated with *Blumeria graminis* f. sp*. hordei* by blowing fresh spores originated from infected barley leaves (∼ 100 conidia/cm^2^). The inoculated leaves were kept on 1% water-agar plates at room temperature under low-light conditions for 2 days.

### *Puccinia graminis* treatment

Urediniospores of *Puccinia graminis* f. sp. *tritici* were collected from infected plants (density of ∼ 10^6^ spores ml^−1^) and sprayed on 10-day-old wheat plants (cv. Bobwhite) that were previously pretreated with oxo-C14-HSL or control (acetone) for 3 days. The inoculated plants were placed for 12 h in the dark. Subsequently, inoculated plants were exposed to normal light condition and kept for 11 days in a growth chamber with an average of 19°C and 90% relative air humidity.

### *Phytophthora infestans* treatment

The *P. infestans* isolate was originally obtained from infected potato foliage. To maintain its virulence, it was invigorated by monthly passage through potato tubers and the *P. infestans* cultures (16–22 days old) were maintained on solid V8 juice agar in the dark at 15°C. In order to obtain *P. infestans* spore solution, the culture was flooded with sterile, distilled water. The spore density was counted using a Fuchs–Rosenthal counting chamber. To improve the zoospore release, the sporangial suspension was placed at 5°C for 3 h and the final solution was adjusted to a density of about 80,000 spores ml^−1^. For the treatment, plants were drenched with the inoculation solution using a pneumatic spray gun and kept at 16°C in the dark with 100% relative air humidity. After 48 h, plants were exposed to a dark/light regime of 16/8 h and 65% relative air humidity. The disease severity was assessed by visual estimation of the infested leaf area and documented with digital pictures. The scale for rating was 1, 5, 10, 20, 30, 50, 60, 70, 80, 90 and 100%. The rating was done 4 and 7 days after inoculation. Each plant was rated separately and means were calculated of five replications per treatment. The formula adapted from Abbott (1925): EF = (Mtr – Mte)/(100 – Mte), in which EF is the percentage of treatment efficiency, Mtr is the percentage of treatment severity and Mte is the percentage of control severity, was used to calculate the efficiency of the treatment.

### *Salmonella* Typhimurium treatment

In order to assess the *Salmonella* proliferation rate in plants, soil-grown 4-week-old *A. thaliana Col*-0 plants were pretreated as indicated before, and thereafter infiltrated using syringe infiltration with wild-type *S. enterica* serovar Typhimurium strain 14028s carrying the pEC75 plasmid conferring resistance to ampicillin. Bacteria were grown until the early log phase in LB medium, washed and resuspended in 10 mM MgCl_2_. Infiltration solution was adjusted to OD_600_ = 0.1, (1.7 × 10^8^ bacteria ml^−1^). Bacterial population was monitored during 6 days post infiltration using selective LB medium containing ampicillin, as described in (Schikora *et al*., 2008).

### DAB staining

Leaves were partially submerged in DAB-staining solution (pH 3.8) at a concentration of 1 mg ml^−1^ for 6 h. Thereafter, leaves were distained with ethanol : chloroform : trichloroacetic acid (4:1:0.15%) solution for 2 days and transferred to 50% glycerol until cytological observations. Development of ESH, formation of papillae or production of ROS were evaluated using an Axioplan 2 (Zeiss, Germany) microscope.

### Transcriptional analyses

Barley cv. Golden Promise leaves pretreated with oxo-C14-HSL or acetone, and subsequently inoculated with *Blumeria graminis* f. sp. *hordei* were harvested at 0, 24 and 48 h after inoculation (hai). Plant material was homogenized and the total RNA was extracted using the Trizol system. cDNA synthesis was perform using 2 μg of total RNA according to qScript cDNA Synthesis Kit (Quanta BioScience Inc.), quantitative RT-PCR was performed using primers listed in Table S1. All expression values were normalized to expression of *HvUBQ60* (Genbank: M60175.1).

### Conclusions

We showed that the resistance-inducing effect of *S. meliloti* in crop plants depends on the production of oxo-C14-HSL. In three different crop plants of worldwide economic importance and relevant for the food chain, oxo-C14-HSL-producing bacteria enhanced their resistance against specific pathogens. In addition, using the *Arabidopsis*–*Salmonella* model, we demonstrate that the same strategy could be a successful method to prevent outbreaks of food-borne diseases originated from plants.

## Supporting information

**Table S1.** List of primers used in quantitative RT-PCR. Annealing temperature for all primers was set at 60°C.
