# Supplementary material for: N-acyl-homoserine lactones-producing bacteria protect plants against plant and human pathogens
Source: Microb Biotechnol. 2014 Sep 19;7(6):580–8. doi: 10.1111/1751-7915.12177 (PMC4265076; doi:10.1111/1751-7915.12177)
Supplement: Supplementary file 1 — Table S1. List of primers used in quantitative RT-PCR. Annealing temperature for all primers was set at 60°C. [file mbt20007-0580-sd1.docx]

# Supplemental Information

## Supplemental Table 1. List of Primers Used in Quantitative RT-PCR

Annealing temperature for all primers was set at 60°C.

| **Gene** | **Primer sequence** |
| --- | --- |
| *HvUBQ* | CAGTAGTGGCGGTCGAAGTG |
|  | ACCCTCGCCGACTACAACAT |
| *HvPR1b* | GGACTACGACTACGGCTCCA |
|  | GGCTCGTAGTTGCAGGTGAT |
| *HvPrx7* | TACCTCTCACATGTCAGCGGC |
|  | TACTACTTCGACCTGATCGCG |
